# Precision measurement of electron-electron scattering in GaAs/AlGaAs using transverse magnetic focusing

**DOI:** 10.1038/s41467-021-25327-7

**Published:** 2021-08-19

**Authors:** Adbhut Gupta, J. J. Heremans, Gitansh Kataria, Mani Chandra, S. Fallahi, G. C. Gardner, M. J. Manfra

**Affiliations:** 1 Department of Physics, Virginia Tech, Blacksburg, VA USA; 2 S-295, New Delhi, Delhi India; 3 Department of Materials Science and Engineering, Rensselaer Polytechnic Institute, Troy, NY USA; 4 Department of Physics and Astronomy, Purdue University, West Lafayette, IN USA; 5 Birck Nanotechnology Center, Purdue University, West Lafayette, IN USA; 6 Microsoft Quantum Purdue, Purdue University, West Lafayette, IN USA; 7 School of Electrical and Computer Engineering, Purdue University, West Lafayette, IN USA; 8 School of Materials Engineering, Purdue University, West Lafayette, IN USA; 9 Present Address: Bradley Department of Electrical and Computer Engineering, Virginia Tech, Blacksburg, VA USA

**Keywords:** Electronic properties and materials, Electronic and spintronic devices

## Abstract

Electron-electron (e-e) interactions assume a cardinal role in solid-state physics. Quantifying the e-e scattering length is hence critical. In this paper we show that the mesoscopic phenomenon of transverse magnetic focusing (TMF) in two-dimensional electron systems forms a precise and sensitive technique to measure this length scale. Conversely we quantitatively demonstrate that e-e scattering is the predominant effect limiting TMF amplitudes in high-mobility materials. Using high-resolution kinetic simulations, we show that the TMF amplitude at a maximum decays exponentially as a function of the e-e scattering length, which leads to a ready approach to extract this length from the measured TMF amplitudes. The approach is applied to measure the temperature-dependent e-e scattering length in high-mobility GaAs/AlGaAs heterostructures. The simulations further reveal current vortices that accompany the cyclotron orbits - a collective phenomenon counterintuitive to the ballistic transport underlying a TMF setting.

## Abstract (web summary)

Electron-electron scattering plays a crucial role in many solid state phenomena; however, the direct measurement of electron-electron scattering length is challenging. Here, the authors use transverse magnetic focusing to measure this quantity in high-mobility GaAs/AlGaAs heterostructures.

## Introduction

Electron–electron (e–e) interactions or scattering play an important role in electronic transport and in solid-state physics in general as they determine the quasiparticle lifetime in a Fermi liquid. Since e–e scattering conserves the total momentum internal to the system we will refer to the e–e scattering as momentum-conserving (MC) scattering in this work. While not affecting mobility due to conservation of total system momentum, in device geometries constructed using two-dimensional electron systems (2DESs) strong MC scattering leads to hydrodynamic phenomena such as vortices^1–14^. The MC scattering time-scale *τ*_MC_, as a fundamental quantity in Fermi liquid theory, has been the subject of several calculations^15–18^. However, direct measurements of *τ*_MC_ have been more elusive, only so far achieved at lower temperature *T* (≲4 K) by loss of quantum interference^19–21^, tunneling measurements^22^, or scattering measurements^23,24^. At higher *T* ≳ 4 K, the effect of MC scattering is to impose a local thermal equilibrium, and here experimental measurements of *τ*_MC_ have only recently been enabled by the hydrodynamic transport regime^11,25^.

We present transverse magnetic focusing (TMF) as a sensitive technique for the measurement of the MC scattering length (${{{{\ell}}}}_{{{{{{{{\rm{MC}}}}}}}}}$) in a 2DES, which for a circular Fermi surface is equivalent to *τ*_MC_. The 2DES is hosted by an ultraclean GaAs/AlGaAs heterostructure grown by optimized MBE process, and is well-suited for this study because the very long momentum-relaxing (MR) scattering length (${{{{\ell}}}}_{{{{{{{{\rm{MR}}}}}}}}}=$ mobility mean-free path ≃ 65 μm at *T* = 4.2 K) provides high sensitivity to MC scattering^11^. MR scattering (mediated by lattice defects and phonons) is responsible for dissipation of the system’s momentum to the lattice. TMF is usually applied to study the ballistic nature of carriers and characterize the shape of the Fermi surface in 3D solids^26,27^ and in 2DESs^28–33^. In the presence of a magnetic field *B* applied normal to the plane of the 2DES, electrons injected from a point contact (PC) follow semiclassical skipping cyclotron orbits of diameter *d*_*c*_ = 2*ℏk*_*F*_/*eB* to focus on a collector PC at a distance *L*_*c*_ = *nd*_*c*_, where *n* is an integer. Here *L*_*c*_ represents the center-to-center distance between injector and collector PCs, *k*_*F*_ the Fermi wavevector, *e* the electron charge, and *ℏ* the Planck’s constant. The nonlocal resistance, defined as the voltage developed at the collector normalized by the injected current, displays maxima at integer *n* due to electrons focusing on the collector. The nonlocal resistance at the maxima, denoted by *R*_*n*_, is largest when all scattering is absent. The decay in *R*_*n*_ with increasing scattering has been observed to be exponential and to possess a characteristic decay length^34,35^, in early work associated with ${{{{\ell}}}}_{{{{{{{{\rm{MR}}}}}}}}}$ (with no reference to ${{{{\ell}}}}_{{{{{{{{\rm{MC}}}}}}}}}$). The increase in scattering can be effectuated by increasing *T* as has been noted in graphene^32,33^. In this work, we address the problem to relate MC scattering and specifically ${{{{\ell}}}}_{{{{{{{{\rm{MC}}}}}}}}}$ to the decay in *R*_*n*_, simultaneously underlining the importance of MC scattering in ballistic transport and introducing a sensitive approach to quantify ${{{{\ell}}}}_{{{{{{{{\rm{MC}}}}}}}}}(T)$.

Using experimentally backed extensive high-resolution kinetic simulations wherein ${{{{\ell}}}}_{{{{{{{{\rm{MC}}}}}}}}}$ is an input parameter, we show that for very long ${{{{\ell}}}}_{{{{{{{{\rm{MR}}}}}}}}}$ the decay in the first peak amplitude (*R*_*n*=1_) due to MC scattering is universal (independent of geometry/device parameters) and obeys

1 $${R}_{n = 1}({d}_{c},{{{{\ell}}}}_{{{{{{{{\rm{MC}}}}}}}}})={R}_{n = 1}({d}_{c},\infty )-{{{\Delta }}}_{n = 1}({d}_{c})\left(1-\exp \left(-\frac{\alpha {d}_{c}}{{{{{\ell}}}}_{{{{{{{{\rm{MC}}}}}}}}}}\right)\right)$$

where *α* is a dimensionless universal parameter characterizing the decay (found below as *α* = 1.34 ± 0.1), *R*_*n*=1_(*d*_*c*_, *∞*) is the *B*-dependent peak amplitude in the ballistic limit (${{{{\ell}}}}_{{{{{{{{\rm{MC}}}}}}}}}\to \infty$), and Δ_*n*=1_(*d*_*c*_) is a *B*-dependent prefactor independent of ${{{{\ell}}}}_{{{{{{{{\rm{MC}}}}}}}}}$, setting the decay due to MC scattering. In accordance with phase-space arguments for temperature scaling of e–e interaction in Fermi liquid theory which lead to ${{{{\ell}}}}_{{{{{{{{\rm{MC}}}}}}}}}\propto {T}^{-2}$, we write:

2 $${{{{\ell}}}}_{{{{{{{{\rm{MC}}}}}}}}}(T)=\alpha {d}_{c}{\left(\frac{{T}_{c}}{T}\right)}^{2}$$

where *T*_*c*_ denotes the (unknown) temperature at which ${{{{\ell}}}}_{{{{{{{{\rm{MC}}}}}}}}}=\alpha {d}_{c}$. Substituting Eq. (2) into Eq. (1) we obtain ${R}_{n = 1}\propto \exp (-{(T/{T}_{c})}^{2})$. Therefore, *T*_*c*_ represents the characteristic temperature scale for decay of *R*_*n*=1_. TMF measurements at variable *T* in our large-scale GaAs/AlGaAs devices will confirm the predicted decay in *R*_*n*=1_, from which we extract *T*_*c*_, ultimately obtaining ${{{{\ell}}}}_{{{{{{{{\rm{MC}}}}}}}}}(T)$. We perform measurements in three devices, each containing several collector PCs placed at distinct *L*_*c*_.

In addition to the known skipping cyclotron orbits along the device boundary, our simulations also reveal accompanying current vortices in the regimes from low to dominant MC scattering. A collective phenomenon such as vortices being observed in a TMF setting, which is widely regarded as a purely ballistic experiment, is counterintuitive. Yet, at *B* = 0 it has been shown both theoretically and experimentally that even ballistic dynamics can lead to collective phenomena^9–11^. Here we show additional evidence in the presence of *B*.

## Results

### TMF devices

**Fig. 1:**
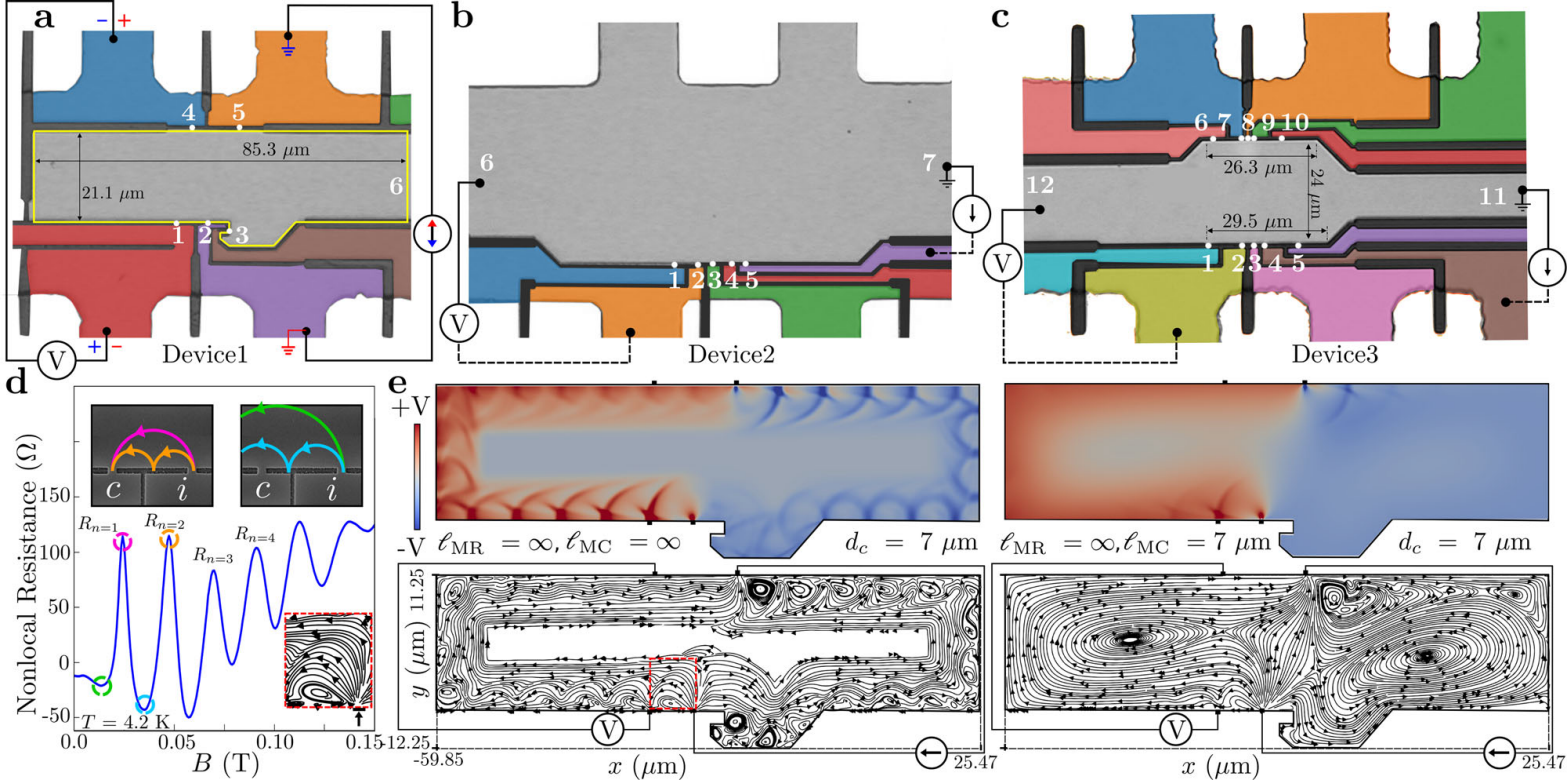
Device geometry and TMF spectra. **a**–**c** Optical images of Device1, Device2, and Device3, respectively, showing dimensions, PCs (indicated by white dots), and measurement configurations with current and voltage PCs marked. The paths leading to the PCs are depicted in different colors for distinct visualization. The computational domain (yellow outline) is indicated for Device1. Device1 features 2 in-line TMF geometries with *L*_*c*_ = 7 μm and 10.5 μm. Device2 features TMF geometries with 7 distinct *L*_*c*_ ranging from 3 to 15 μm. Device3 features TMF geometries with 10 distinct *L*_*c*_ ranging from 1.3 to 20.5 μm (data from 6 geometries each in Device2 and Device3 are presented, omitting closely spaced *L*_*c*_). **d** TMF spectrum (nonlocal resistance vs *B* in Device1, *L*_*c*_ = 7 μm) = *V*_1,4_/*I*_2,5_ (where *V*_1,4_ = *V*_1_−*V*_4_ and *I*_2,5_ is the conventional current from 2 to 5 inside the device) as obtained in experiment at *T* = 4.2 K. The inset shows schematics of semiclassical cyclotron orbits corresponding to first two maxima and minima, indicated using the same color in the TMF spectrum. Maxima (*R*_*n*_) occur at *L*_*c*_ = *nd*_*c*_ where integer *n* represents the number of orbits electrons follow before focusing into *c* (*n* indicated on the corresponding maxima). Minima occur at values slightly less than half-integer *n*. **e** Simulated voltage contour plots (top) and current streamlines (bottom) in Device1 for the first maximum (*d*_*c*_ = 7 *μ*m) for ${{{{\ell}}}}_{{{{{{{{\rm{MC}}}}}}}}}\to \infty$ (left) and finite ${{{{\ell}}}}_{{{{{{{{\rm{MC}}}}}}}}}$ (right). Both show cyclotron orbits and vortices with distinct voltage and current patterns, which for ${{{{\ell}}}}_{{{{{{{{\rm{MC}}}}}}}}}\to \infty$ are highlighted in the dashed red box and are magnified in the inset of **d** (cfr text: ${{{{\ell}}}}_{{{{{{{{\rm{MR}}}}}}}}}\to \infty$ throughout most simulations).

The TMF geometries were patterned on a 2DES in a quantum well in an ultraclean GaAs/AlGaAs heterostructure of mobility *μ* exceeding 670 m^2^V^−1^s^−1^ at *T* = 4.2 K across all devices (Fig. 1a). At the areal electron density *N*_*s*_ ≈ 3 × 10^15^ m^−2^, the Fermi energy *E*_*F*_ ≈ 10.9 meV. The methods and transport properties are described in Supplementary Notes 1 and 2. Experiments were performed on multiprobe Hall mesas in three devices—Device1, Device2, and Device3^11^ bearing numerous in-line TMF geometries with *L*_*c*_ ranging from 7–10.5 μm in Device1, 3–15 μm in Device2, and 1.3–20.5 μm in Device3 (Fig. 1a–c). Each TMF geometry features two PCs which can act either as an injector or collector. The conducting width of each PC is *w* ≈ 0.6 μm and the Fermi wavelength, *λ*_*F*_ ≈ 43 nm implying that *w*/(*λ*_*F*_/2) ≈ 28 spin-degenerate transverse modes contribute to transport, yielding a PC resistance ≈ (*h*/2*e*^2^)/28 = 461 Ω. The large number of modes indicate that quantized transport through the PC apertures can be neglected. Measurements were performed in the linear response regime at 4.2 K < *T* < 36 K, using low-frequency lock-in techniques without any DC offsets, and under a small excitation current *I* ∼ 100–200 nA to avoid electron heating. The boundaries of the device were defined by wet etching, resulting in specular scattering at the boundaries^30,36^.

An example of experimental results is depicted in Fig. 1d for *L*_*c*_ = 7 μm in Device1 depicting the untreated nonlocal resistance at the collector vs *B* (referred to as TMF spectra) at 4.2 K. The maxima originate from cyclotron orbits impinging in the vicinity of, or directly on, the collector (*c* in inset of Fig. 1d). For the minima, the orbits straddle the collector (inset of Fig. 1d).

### TMF simulations

We simulate magnetotransport in the actual experimental device geometry using BOLT, a high-resolution solver for kinetic theories^9,11,37^, which solves the Boltzmann transport equation:

3 $$\frac{1}{{v}_{F}}\frac{\partial f}{\partial t}+\left(\frac{{{{{{{{\bf{p}}}}}}}}}{m{v}_{F}}\right).\frac{\partial f}{\partial {{{{{{{\bf{x}}}}}}}}}+\left(\frac{2}{{d}_{c}}\right)\frac{\partial f}{\partial \theta }=-\frac{f-{{f}_{0}}^{{{{{{{{\rm{MR}}}}}}}}}}{{{{{\ell}}}}_{{{{{{{{\rm{MR}}}}}}}}}}-\frac{f-{{f}_{0}}^{{{{{{{{\rm{MC}}}}}}}}}}{{{{{\ell}}}}_{{{{{{{{\rm{MC}}}}}}}}}}$$

where *f*(**x**, **p**, *t*) is the probability distribution of electrons along the spatial coordinates **x** ≡ (*x*, *y*) and momentum coordinates ${{{{{{{\bf{p}}}}}}}}\equiv \hslash {k}_{F}(\cos (\theta ),\sin (\theta ))$, where *θ* denotes the angle on the Fermi surface, *t* denotes time, and *v*_*F*_ denotes the Fermi velocity. The Lorentz force due to *B* appears in the third term on the left, which for a circular Fermi surface simplifies to the form shown. Long-range electric fields are not explicitly included, but their effects are accounted for at linear order through a renormalized chemical potential^9^. The injected particles, with an effective mass *m*, are constrained to remain on the Fermi surface, move at *v*_*F*_, and are injected over all angles following a cos(*θ*) distribution which is maximum perpendicular to the boundary into which the contact is placed^10^. The effect of the RHS of Eq. (3) is a thermalization of carriers to local stationary and drifting Fermi-Dirac distributions, ${f}_{0}^{{{{{{{{\rm{MR}}}}}}}}}$ and ${f}_{0}^{{{{{{{{\rm{MC}}}}}}}}}$, due to MR and MC scattering, respectively. This is implemented using a dual relaxation time approximation with scattering length scales ${{{{\ell}}}}_{{{{{{{{\rm{MR}}}}}}}}}$ and ${{{{\ell}}}}_{{{{{{{{\rm{MC}}}}}}}}}$. Further details regarding the collision operators are given in ref. ^9^. We consider perfectly reflecting device boundaries, with carriers injected by imposing a shifted Fermi-Dirac distribution at the locations of the contacts.

**Fig. 2:**
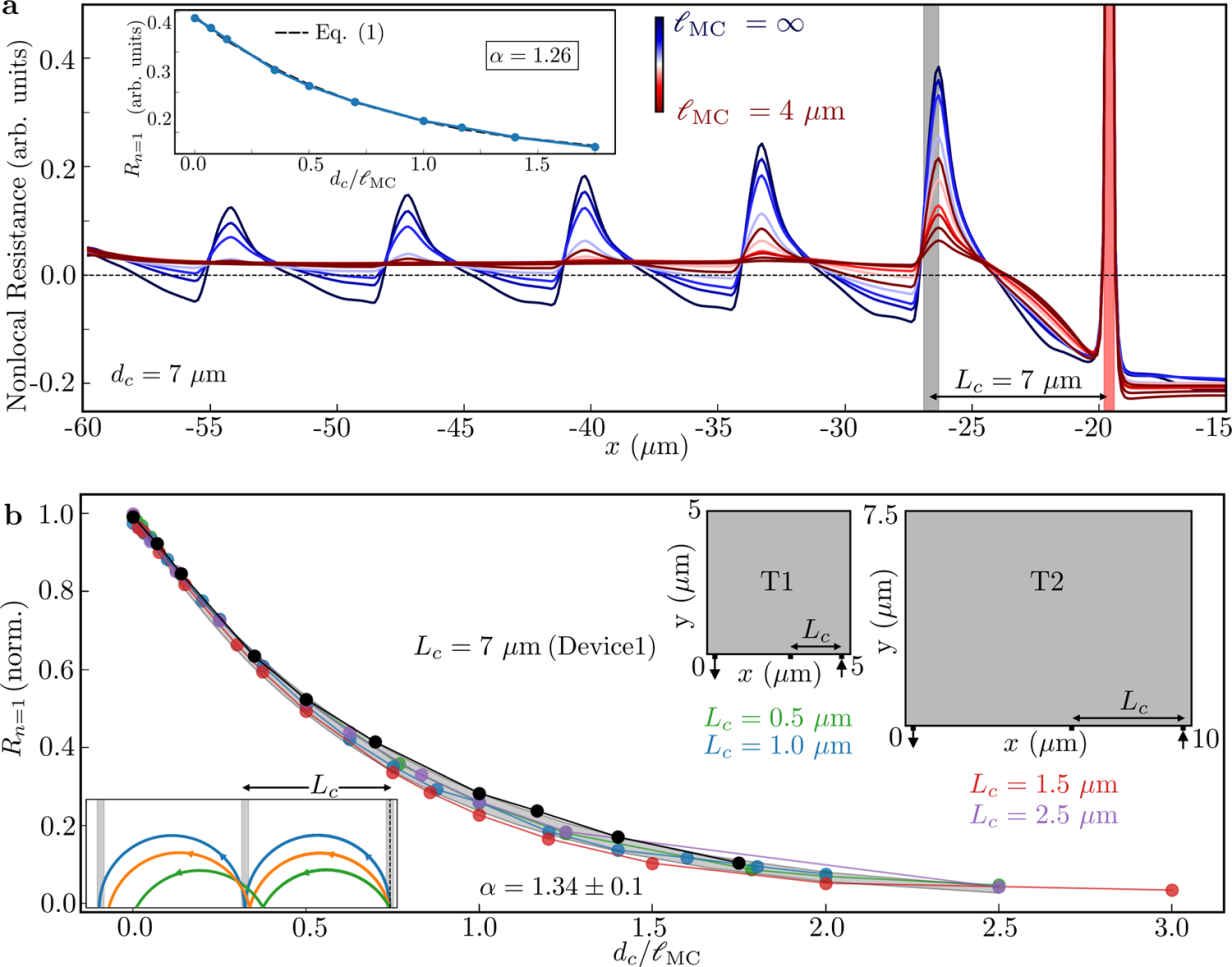
Universal decay of TMF amplitude. **a** Simulated nonlocal resistance in Device1 (Fig. 1a, e, *L*_*c*_ = *d*_*c*_ = 7 μm) plotted vs position *x* (corresponding to Fig. 1e) along the edge of the device into which the injection PC (red vertical bar) is placed, for various values of ${{{{\ell}}}}_{{{{{{{{\rm{MC}}}}}}}}}$ starting from ${{{{\ell}}}}_{{{{{{{{\rm{MC}}}}}}}}}\to \infty$ to progressively smaller values. The grey bar indicates the position of collector PC. Inset shows the decay of the first maximum as a function of ${d}_{c}/{{{{\ell}}}}_{{{{{{{{\rm{MC}}}}}}}}}$, along with a fit to Eq. (1) (black dotted line). **b** The normalized *R*_*n*=1_ plotted vs parameter ${d}_{c}/{{{{\ell}}}}_{{{{{{{{\rm{MC}}}}}}}}}$ for T1 (*L*_*c*_ = *d*_*c*_ = 0.5, 1.0 μm; variable ${{{{\ell}}}}_{{{{{{{{\rm{MC}}}}}}}}}$), T2 (*L*_*c*_ = *d*_*c*_ = 1.5, 2.5 μm; variable ${{{{\ell}}}}_{{{{{{{{\rm{MC}}}}}}}}}$), and Device1 (*L*_*c*_ = *d*_*c*_ = 7 μm; variable ${{{{\ell}}}}_{{{{{{{{\rm{MC}}}}}}}}}$). The normalized curves for all the devices nearly overlap revealing that *R*_*n*=1_ follows an exponential decay vs ${d}_{c}/{{{{\ell}}}}_{{{{{{{{\rm{MC}}}}}}}}}$ independent of geometry, with universal *α* = 1.34 ± 0.1. Test devices T1 and T2 are depicted as insets with values of *L*_*c*_ = *d*_*c*_ simulated for each device. The evolution of the current streamlines as we vary ${{{{\ell}}}}_{{{{{{{{\rm{MC}}}}}}}}}$ is depicted in Supplementary Note 3 for T1 for *L*_*c*_ = *d*_*c*_ = 1.0 μm. The inset depicts the defocusing of electrons injected at angles different from 90^∘^ (green and orange trajectories) resulting in lesser number of electrons focusing exactly at *nd*_*c*_ with each reflection and in a decrease in maximum amplitude despite specular boundary reflection.

The simulations are performed in two simplified test geometries T1 and T2, and in the complicated experimental geometries. To initially isolate the effects of ${{{{\ell}}}}_{{{{{{{{\rm{MC}}}}}}}}}$ on TMF spectra, we set ${{{{\ell}}}}_{{{{{{{{\rm{MR}}}}}}}}}\to \infty$, neglect Fermi surface thermal smearing, and treat ${{{{\ell}}}}_{{{{{{{{\rm{MC}}}}}}}}}$ as a free parameter. We show later that the exceptionally long ${{{{\ell}}}}_{{{{{{{{\rm{MR}}}}}}}}}$ in our 2DES has a minimal effect on the TMF spectra and that the effect of Fermi surface smearing on *R*_*n*=1_ is negligible. We start in Fig. 1e, left panels with the limiting ballistic case where ${{{{\ell}}}}_{{{{{{{{\rm{MC}}}}}}}}}\to \infty$, and consider Device1 with *d*_*c*_ set to *L*_*c*_ = 7 μm (fixed *B*). As expected from single-particle insight, carriers emanating from the injector propagate along skipping orbits on the bottom edge, under the influence of Lorentz force. Interestingly, current vortices accompany these cyclotron orbits, even in the absence of all microscopic interactions. This observation reinforces the existence of collective phenomena in the ballistic transport regime, as highlighted in recent work^9–11^. Next, in Fig. 1e, right panels we approach the hydrodynamic regime by setting ${{{{\ell}}}}_{{{{{{{{\rm{MC}}}}}}}}}={d}_{c}=7$μm, and observe a profound change in the voltage contour and current streamline plots. A large vortex inhabits the main chamber and displaces most of the cyclotron orbits except orbits near the injector. Notably, the vortices in the hydrodynamic regime are distinct from those in the ballistic regime^11^. The ballistic regime exhibits multiple vortices of various scales and at various locations in the device even in the absence of electron–electron interactions (see left panel of Fig. 1e and Supplementary Fig. 4), while the dominance of electron–electron scattering in the hydrodynamic regime favors large device-scale vortices (see right panel of Fig. 1e and Supplementary Fig. 3). When plotting the simulated nonlocal resistance vs position *x* along the bottom edge of the device, for fixed *B* (corresponding to *d*_*c*_ = *L*_*c*_ = 7 μm) for various ${{{{\ell}}}}_{{{{{{{{\rm{MC}}}}}}}}}$ (Fig. 2a), the role of MC scattering in limiting the TMF signal becomes apparent. Two additional observations appear from Fig. 2a. First, the maxima occur at distances slightly below *L*_*c*_ = *nd*_*c*_, with a deviation of ≲5% (experimentally leading to TMF maxima slightly below the expected *B* = *n*2*ℏk*_*F*_/*eL*_*c*_^31^). Second, *R*_*n*_ decreases with increasing *n* despite perfect specular boundary scattering. These phenomena result from the angular distribution of injected electrons. The electrons injected at an angle different from 90^∘^, lead to a defocusing effect such that with each reflection off the boundary, the number of electrons focusing at precisely *nd*_*c*_ decreases, leading to a decrease in *R*_*n*_ with increasing *n* (*R*_*n*=1_ > *R*_*n*=2_ > . . . ). This suggests that the ratio of subsequent TMF maxima values *R*_*n*_, is not a good measure to infer specularity of the boundary as has been used by various works^32,33,38^.

### Extraction of ${\ell}_{{{{{{{{\rm{MC}}}}}}}}}$ using TMF

We next extract the functional dependence of *R*_*n*=1_ on ${\ell}_{{{{{{{{\rm{MC}}}}}}}}}$. Varying ${{{{\ell}}}}_{{{{{{{{\rm{MC}}}}}}}}}$ in the simulations, we plot *R*_*n*=1_ for the various geometries considered versus the parameter *d*_*c*_/${{{{\ell}}}}_{{{{{{{{\rm{MC}}}}}}}}}$. In Fig. 2b we find a data collapse to the function ${R}_{n = 1}({d}_{c},{{{{\ell}}}}_{{{{{{{{\rm{MC}}}}}}}}})\propto \exp (-\alpha {d}_{c}/{{{{\ell}}}}_{{{{{{{{\rm{MC}}}}}}}}})$, where *α* = 1.34 ± 0.1 is a dimensionless parameter independent of the device geometry, *B* or energy dispersion. However, *α* may depend on the shape of the Fermi surface, restricted in our simulations to a circle. Using the maximal value *R*_*n*=1_(*d*_*c*_, *∞*) in the ballistic limit (${{{{\ell}}}}_{{{{{{{{\rm{MC}}}}}}}}}\to \infty$), we obtain the form of Eq. (1), with Δ_*n*=1_(*d*_*c*_) independent of ${{{{\ell}}}}_{{{{{{{{\rm{MC}}}}}}}}}$. We note that *R*_*n*=1_ from Eq. (1) is independent of device geometry because it is a local quantity if *d*_*c*_ ≪ *W* with *W* the device scale or open distance in any direction away from the PC. For validity of Eq. (1) we require ${k}_{F}^{-1}\ll w\ll {d}_{c}\ll W$. The condition ${k}_{F}^{-1}\ll w$ ensures that sufficient modes are injected into the system for semiclassical transport. The condition *w* ≪ *d*_*c*_ ensures that magnetic quantization effects are absent. The condition *d*_*c*_ ≪ *W* avoids unwanted boundary scattering, ensuring that skipping orbits reach the collector without hindrance from device boundaries in any direction. We show in the Supplementary Note 4 that when *d*_*c*_ ∼ *W*, the decay is no longer universal since carriers now sense the device boundaries. In contrast to *R*_*n*_, the nonlocal resistance at *B* = 0 in the ballistic and hydrodynamic regimes as measured in several recent experiments^7,8,11^, is sensitive to the nonlocal current–voltage relation of the underlying regime, and therefore depends on the device geometry.

**Fig. 3:**
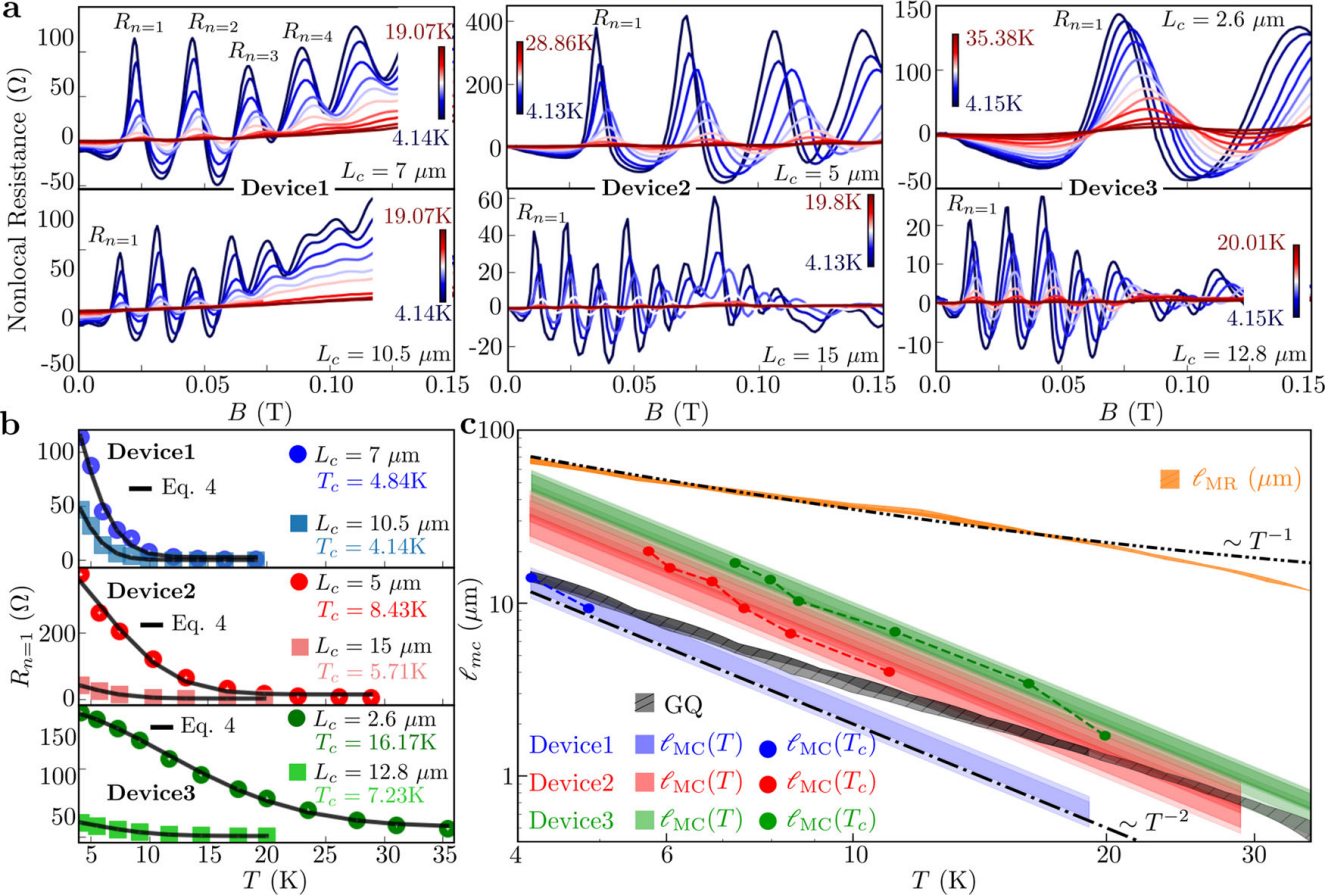
Dependence on *T* of TMF and extraction of ${{{{\ell}}}}_{{{{{{{{\rm{MC}}}}}}}}}$. **a** TMF spectra for specified *L*_*c*_ in Device1, Device2, and Device3 over indicated range of *T* (dependent on *L*_*c*_ and device). Increasing *T* causes two effects—suppression of TMF and a shift in location of the maxima along *B*. The shift in *B* is caused by *N*_*s*_ increasing with *T* as discussed in Supplementary Note 2. **b** First maximum amplitude *R*_*n*=1_ plotted as a function of *T* for the spectra depicted in **a**. The black solid lines represent a fit to Eq. (4), demonstrating the dominant effect of MC scattering on TMF. **c** Measured ${{{{\ell}}}}_{{{{{{{{\rm{MC}}}}}}}}}({T}_{c})$ plotted vs *T*_*c*_ for Device1 (blue), Device2 (red), and Device3 (green) on double-logarithmic scale. The shaded regions depict the calculated values using Eq. (2) plotted vs *T* with error bars. The grey region labeled GQ represents the theoretical value of ${{{{\ell}}}}_{{{{{{{{\rm{MC}}}}}}}}}$ from Eq. (5) for comparison. A reference line *T*^−2^ is drawn as a guide to the eye emphasizing that experimental ${{{{\ell}}}}_{{{{{{{{\rm{MC}}}}}}}}}({T}_{c})$ decay with *T*^−2^. Experimental ${{{{\ell}}}}_{{{{{{{{\rm{MR}}}}}}}}}$ vs *T* (orange) is plotted with a reference line depicting the expected *T*^−1^ fall off. ${{{{\ell}}}}_{{{{{{{{\rm{MR}}}}}}}}} \, > \, {{{{\ell}}}}_{{{{{{{{\rm{MC}}}}}}}}}$ and ${{{{\ell}}}}_{{{{{{{{\rm{MR}}}}}}}}} > {L}_{c}$ throughout the experiments indicating the minimal effect of ${{{{\ell}}}}_{{{{{{{{\rm{MR}}}}}}}}}$ on TMF.

The universal decay of ${R}_{n = 1}({d}_{c},{{{{\ell}}}}_{{{{{{{{\rm{MC}}}}}}}}})$ provides an opportunity to measure ${{{{\ell}}}}_{{{{{{{{\rm{MC}}}}}}}}}$ experimentally. Figure 3a depicts the experimental TMF spectra measured at various *T* for selected *L*_*c*_ in Device1 (*L*_*c*_ = 7 μm and 10.5 μm), Device2 (*L*_*c*_ = 5 μm and 15 μm) and Device3 (*L*_*c*_ = 2.6 μm and 12.8 μm). Measurements in other geometries can be found in Supplementary Note 5. We first convert the universal ${R}_{n = 1}({d}_{c},{{{{\ell}}}}_{{{{{{{{\rm{MC}}}}}}}}})$ into *R*_*n*=1_(*d*_*c*_, *T*), the quantity measured in experiments. Substituting ${{{{\ell}}}}_{{{{{{{{\rm{MC}}}}}}}}}(T)$ of Eq. (2) into Eq. (1), we obtain:

4 $${R}_{n = 1}({d}_{c},T)={R}_{n = 1}({d}_{c},0)-{{{\Delta }}}_{n = 1}({d}_{c})\left(1-\exp \left(-{\left(\frac{T}{{T}_{c}}\right)}^{2}\right)\right)$$

Equation (4) represents a model for *R*_*n*=1_(*d*_*c*_, *T*) to which experimental data can be fit with three fitting parameters—*R*_*n*=1_(*d*_*c*_, 0), Δ_*n*=1_(*d*_*c*_), and most importantly, *T*_*c*_. Knowing *T*_*c*_ allows determination of ${{{{\ell}}}}_{{{{{{{{\rm{MC}}}}}}}}}(T={T}_{c})=(1.34\pm 0.1){d}_{c}$. By measuring *R*_*n*=1_(*d*_*c*_, *T*) at various *L*_*c*_ = *d*_*c*_ (each *L*_*c*_ corresponding to one *T*_*c*_), one can obtain ${{{{\ell}}}}_{{{{{{{{\rm{MC}}}}}}}}}$ at a series of temperatures *T* = *T*_*c*_. We note that measuring *R*_*n*=1_(*d*_*c*_, *T*) for different *T* at fixed *L*_*c*_ = *d*_*c*_ yields ${{{{\ell}}}}_{{{{{{{{\rm{MC}}}}}}}}}$ at a single *T* = *T*_*c*_. Values for ${{{{\ell}}}}_{{{{{{{{\rm{MC}}}}}}}}}(T)$ for different *T* can then be calculated by Eq. (2), but such values do not constitute a direct measurement. The scheme offers a straightforward interpretation in that measurements at the length scale *L*_*c*_ probe e–e scattering at the energy scale set by *T*_*c*_. Figure 3b depicts the experimental values for maxima *R*_*n*=1_(*d*_*c*_, *T*) in Device1, Device2, and Device3 for *L*_*c*_ = *d*_*c*_ of Fig. 3a. Notably, Fig. 3b shows that Eq. (4) is closely obeyed, providing evidence that Eq. (2) not only represents the most straightforward form compatible with phase-space arguments for a 2DES in a GaAs/AlGaAs heterostructure, but also is closely followed. Recent TMF experiments in graphene^32,33^ have likewise obtained similar dependence on *T* of maxima *R*_*n*_. For every *L*_*c*_, Fig. 3b yields corresponding *T*_*c*_ and ${{{{\ell}}}}_{{{{{{{{\rm{MC}}}}}}}}}({T}_{c})=(1.34\pm 0.1){d}_{c}$, plotted in Fig. 3c for all devices. Using Eq. (2), we can calculate the values of ${{{{\ell}}}}_{{{{{{{{\rm{MC}}}}}}}}}$ for continuous *T*. In Fig. 3c we overplot ${{{{\ell}}}}_{{{{{{{{\rm{MC}}}}}}}}}(T)$ calculated by Eq. (2) such that the shaded regions depict the possible range of ${{{{\ell}}}}_{{{{{{{{\rm{MC}}}}}}}}}$ for a particular device (see Supplementary Note 7 for sources of uncertainty in the calculation of ${{{{\ell}}}}_{{{{{{{{\rm{MC}}}}}}}}}$). Figure 3c shows that the data for ${{{{\ell}}}}_{{{{{{{{\rm{MC}}}}}}}}}({T}_{c})$ indeed decays as ${T}_{c}^{-2}$ (in accordance with Fermi liquid theory) which is an expected but nontrivial finding because rather than being an assumption it now stems from a direct measurement. Since the quantities *R*_*n*=1_(*d*_*c*_, 0) and Δ_*n*=1_(*d*_*c*_) in Eq. (4) only depend on *B* and not on *T*, they can be eliminated and a closed form expression can be obtained for *T*_*c*_ as derived in Supplementary Note 6.

## Discussion

The same slopes but different intercepts of ${{{{\ell}}}}_{{{{{{{{\rm{MC}}}}}}}}}$ vs *T* in Fig. 3c show that in Device1, Device2, and Device3, ${{{{\ell}}}}_{{{{{{{{\rm{MC}}}}}}}}}$ follows a dependence *T*^−2^, but with a multiplicative prefactor dependent on device. We surmise that the nonuniversal prefactor originates in device-dependent electrostatic environments for the 2DES. While all three devices are fabricated on nominally the same heterostructure, the 3D electrostatic environment of the 2DES can vary between devices due to residual charged impurities, leading to varying levels of dielectric screening^39^. We then expect ${{{{\ell}}}}_{{{{{{{{\rm{MC}}}}}}}}}$ to possess a nonuniversal prefactor dependent on the screening strength^39^, affecting the magnitude of ${{{{\ell}}}}_{{{{{{{{\rm{MC}}}}}}}}}$ but not the dependence *T*^−2^. In Fig. 3c, the extracted ${{{{\ell}}}}_{{{{{{{{\rm{MC}}}}}}}}}$ is compared to values predicted by a commonly used theoretical expression^15^:

5 $${{{{\ell}}}}_{{{{{{{{\rm{MC}}}}}}}}}^{-1}=\frac{{({k}_{B}T)}^{2}}{h{E}_{F}{v}_{F}}\left\{{{{{{{\mathrm{ln}}}}}}}\,\frac{{E}_{F}}{{k}_{B}T}+{{{{{{\mathrm{ln}}}}}}}\,\frac{2{q}_{{{{{{{{\rm{TF}}}}}}}}}}{{k}_{F}}+1\right\}$$

where *q*_TF_ represents the Thomas-Fermi wavevector and *k*_*B*_ the Boltzmann constant. The values of *T*, *E*_*F*_, *v*_*F*_, *k*_*F*_, and *q*_TF_ are obtained from experiment. Agreement is observed between the data and calculated ${{{{\ell}}}}_{{{{{{{{\rm{MC}}}}}}}}}$ and Eq. (5) in Device1 at lower *T*. However in Device2 and Device3 we observe longer ${{{{\ell}}}}_{{{{{{{{\rm{MC}}}}}}}}}$ than predicted by Eq. (5), especially at lower *T*, and in all three devices the dependence on *T* predicted by Eq. (5) deviates from experiment. Again dielectric screening plays a role in the deviation. While many-body screening due to electrons is accounted for in Eq. (5) using the random phase approximation (RPA), dielectric screening is not included, as noted in ref. ^39^. Varying levels of dielectric screening and concomitantly varying 3D electrostatic environments hence explain the nonuniversal prefactor of ${{{{\ell}}}}_{{{{{{{{\rm{MC}}}}}}}}}$ as well as the deviation of Eq. (5) from experiment. Notably, measurements of ${{{{\ell}}}}_{{{{{{{{\rm{MC}}}}}}}}}$ obtained from nonlocal resistance measurements at *B* = 0 in Device3^11^, yield similar values of ${{{{\ell}}}}_{{{{{{{{\rm{MC}}}}}}}}}$ as presented here, albeit with larger error bars.

**Fig. 4:**
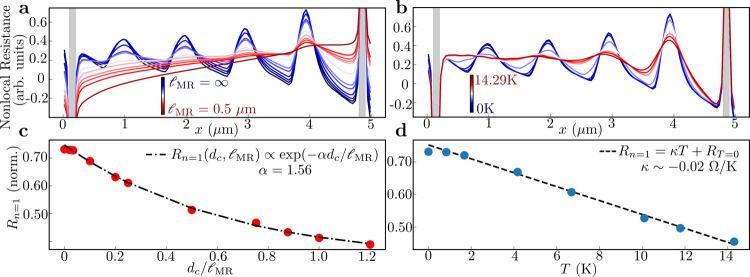
Decay of TMF with MR scattering and thermal Fermi surface broadening. **a**, **b** Simulated nonlocal resistance in T1 plotted versus position *x* along the edge of the device into which the injection PC is placed, for variable ${{{{\ell}}}}_{{{{{{{{\rm{MR}}}}}}}}}$ from ${{{{\ell}}}}_{{{{{{{{\rm{MR}}}}}}}}}\to \infty$ to 0.5 μm in **a**, and for variable *T* from *T* → 0 K to *T* = 14.3 K in **b**. **c** Simulated ${R}_{n = 1}({d}_{c},{{{{\ell}}}}_{{{{{{{{\rm{MR}}}}}}}}})$ assuming ${{{{\ell}}}}_{{{{{{{{\rm{MC}}}}}}}}}\to \infty$, plotted vs ${d}_{c}/{{{{\ell}}}}_{{{{{{{{\rm{MR}}}}}}}}}$ revealing an exponential decay with decay constant *α* = 1.56. **d** Simulated *R*_*n*=1_ assuming ${{{{\ell}}}}_{{{{{{{{\rm{MR}}}}}}}}},{{{{\ell}}}}_{{{{{{{{\rm{MC}}}}}}}}}\to \infty$, plotted vs *T* along with a linear fit (dashed line). Both MR scattering and thermal Fermi surface broadening play a lesser role in the decay of TMF in the experiments.

We next address the effects on the decay of the TMF maxima of MR scattering due to impurity scattering and phonons, and of Fermi surface broadening under finite *T* (Fig. 4) (broadening by injection energy has also been considered^35,40^). We first discuss MR scattering. Repeating the simulations, but now with only MR scattering present (variable ${{{{\ell}}}}_{{{{{{{{\rm{MR}}}}}}}}}$, ${{{{\ell}}}}_{{{{{{{{\rm{MC}}}}}}}}}\to \infty$), in Fig. 4a, c we find that ${R}_{n = 1}({d}_{c},{{{{\ell}}}}_{{{{{{{{\rm{MR}}}}}}}}})$ follows the same exponential dependence as observed for MC scattering, albeit with a different *α* = 1.56. While *α* is expected to be order ${{{{{{{\mathcal{O}}}}}}}}(\pi /2)$ as indeed borne out, the precise values for *α* follow simulation results. The same functional dependence is not surprising, since both MR and MC scattering result in the same physical process affecting TMF: randomization of individual carrier trajectories, leading to defocusing. The difference in *α* suggests that for same values of ${{{{\ell}}}}_{{{{{{{{\rm{MC}}}}}}}}}$ and ${{{{\ell}}}}_{{{{{{{{\rm{MR}}}}}}}}}$, the decay is slightly more sensitive to MC scattering than to MR scattering. However, throughout the range of *T* in our experiment we have ${{{{\ell}}}}_{{{{{{{{\rm{MR}}}}}}}}} \, > \, {{{{\ell}}}}_{{{{{{{{\rm{MC}}}}}}}}}$ and ${{{{\ell}}}}_{{{{{{{{\rm{MR}}}}}}}}} \, > \, {L}_{c}$ due to the ultraclean heterostructure, and hence ${{{{\ell}}}}_{{{{{{{{\rm{MR}}}}}}}}}$ has minimal impact on the TMF spectra. This is corroborated in Fig. 3c, showing that the decay of *R*_*n*=1_(*T*) vs *T* is not consistent with the measured ${{{{\ell}}}}_{{{{{{{{\rm{MR}}}}}}}}}(T)$ vs *T* (obtained from standard 4-probe measurement of mobility and *N*_*s*_). Figure 3c compares ${{{{\ell}}}}_{{{{{{{{\rm{MC}}}}}}}}}$ to ${{{{\ell}}}}_{{{{{{{{\rm{MR}}}}}}}}}$ and clearly shows ${{{{\ell}}}}_{{{{{{{{\rm{MC}}}}}}}}} \, < \, {{{{\ell}}}}_{{{{{{{{\rm{MR}}}}}}}}}$, ensuring the consistency of Eq. (1) wherein MR scattering is ignored. We note that ${{{{\ell}}}}_{{{{{{{{\rm{MR}}}}}}}}}$ needs to be compared against *L*_*c*_ and not *W*, while in contrast ballistic phenomena at *B* = 0 require ${{{{\ell}}}}_{{{{{{{{\rm{MR}}}}}}}}} \, > \, W$^11^. Therefore, the effect of MR scattering can be negated by choosing a sufficiently small *L*_*c*_, which also allows for measurements of ${{{{\ell}}}}_{{{{{{{{\rm{MC}}}}}}}}}$ using higher *T*_*c*_.

To identify the effect of thermal broadening of the Fermi surface on the TMF signal, we perform ideal ballistic simulations (${{{{\ell}}}}_{{{{{{{{\rm{MR}}}}}}}}},{{{{\ell}}}}_{{{{{{{{\rm{MC}}}}}}}}}\to \infty$) at finite *T*. As indicated in Fig. 4b, d, thermal smearing merely produces a linear decay in the TMF amplitude, and is thus a subordinate effect compared to the effect of MR and particularly MC scattering.

A surprising finding from the kinetic simulations lies in the presence of collective phenomena in a ballistic TMF setup—the formation of current vortices between the injector and collector probes (Fig. 1e), which causes a local enhancement in the magnetic field. Current vortices, usually associated with hydrodynamic intuition, have only recently been associated with the ballistic regime at *B* = 0^9–11^, and the present work shows vortices can occur at finite *B* as well. The vortices cannot be understood by examining individual single-particle trajectories, but rather appear as collective phenomena of all the particles as a whole, in currents resulting from a vector sum over all the trajectories. While TMF has seen extensive numerical study^41–46^, current vortices have not been reported till this work. A probable reason lies in the observation that current computation may be dominated by shot noise in TMF simulations performed using particle schemes^44,45^, a limitation not suffered by the present high-resolution kinetic scheme. It would be interesting to check if vortices persist in the coherent transport regime^43,46^.

To conclude, by combined experiments and high-resolution kinetic simulations we show that transverse magnetic focusing amplitudes decay exponentially due to electron–electron scattering, demonstrating the more general importance of electron–electron scattering in ballistic transport in high-mobility materials. Analysis of the transverse magnetic focusing amplitudes thereby allows for a precision measurement of the electron–electron scattering length, of importance in solid-state systems. The kinetic simulations reveal the hitherto unsuspected presence of current vortices even in a ballistic transverse magnetic focusing setup.

## Methods

The geometries were patterned by gently wet etching of the GaAs/AlGaAs heterostructure in H_2_SO_4_/H_2_O_2_/H_2_O solution after electron beam lithography, using PMMA as etching mask, to a depth removing the GaAs quantum well hosting the 2DES. Prior to electron-beam lithography, a Hall mesa was defined by photolithography and wet etching in the same solution. Ohmic contacts were annealed InSn. Measurements were performed at 4.2 K < *T* < 36 K in a sample-in-exchange-gas system, using low frequency (~45 Hz) lock-in techniques under AC current bias without DC offsets. The transport properties of the unpatterned material were independently characterized on a sample in the van der Pauw geometry using the same methods but omitting lithography steps.

## Supplementary information

Supplementary Information

## Data Availability

The data that support the plots within this paper and other findings of this study are available from the corresponding author upon reasonable request.

## Code availability

The source code for the simulations can be found at https://www.github.com/mchandra/bolt.
