# Supplementary material for: Precision measurement of electron-electron scattering in GaAs/AlGaAs using transverse magnetic focusing
Source: Nat Commun. 2021 Aug 19;12:5048. doi: 10.1038/s41467-021-25327-7 (PMC8376939; doi:10.1038/s41467-021-25327-7)
Supplement: Supplementary file 1 — Supplementary Information [file 41467_2021_25327_MOESM1_ESM.pdf]

Supplementary Information

Precision measurement of electron-electron scattering in GaAs/AlGaAs using Transverse Magnetic Focusing

Adbhut Gupta,[1] J. J. Heremans,[1,*] Gitansh Kataria,[2,†] Mani Chandra,[3] S. Fallahi,[4,5] G. C. Gardner,[5,6] and M. J. Manfra[4,5,6,7,8]

[1]*Department of Physics, Virginia Tech, Blacksburg, Virginia 24061, USA*

[2]*S-295, Greater Kailash 2, New Delhi, Delhi 110048, India*

[3]*Department of Materials Science and Engineering, Rensselaer Polytechnic Institute, Troy, New York 12180, USA*

[4]*Department of Physics and Astronomy, Purdue University, West Lafayette, Indiana 47907, USA*

[5]*Birck Nanotechnology Center, Purdue University, West Lafayette, Indiana 47907, USA*

[6]*Microsoft Quantum Purdue, Purdue University, West Lafayette, Indiana 47907, USA*

[7]*School of Electrical and Computer Engineering, Purdue University, West Lafayette, Indiana 47907, USA*

[8]*School of Materials Engineering, Purdue University, West Lafayette, Indiana 47907, USA*

* heremans@vt.edu

† Future address: Bradley Department of Electrical and Computer Engineering, Virginia Tech, Blacksburg, Virginia 24061, USA

Supplementary Note 1. Device fabrication and material properties

The mesoscopic geometries were patterned using electron beam lithography followed by wet etching of the barriers, using PMMA as the etching mask. Each device contains several mesoscopic apertures (point contacts, PCs) separated by various distances L_c. Each PC can function either as current injector i or collector (voltage detector) c. The PC resistance R_{pc} varies between 450 Ω to 750 Ω at $T = 4.2$ K, depending on the PC and device. The devices were fabricated from GaAs/AlGaAs MBE-grown material hosting the two-dimensional electron system (2DES). The GaAs quantum well is located 190 nm below the surface, has a width of 26 nm, and is top- and bottom-doped by Si $\delta-$layers 80 nm removed from the quantum well and embedded in $Al_{0.32}Ga_{0.68}As$ barriers. Optimization of heterostructure design is described in Supplementary Ref.[1].

The van der Pauw method was used to characterize electron transport properties of the unpatterned 2DES. The values of 2D resistivity $R_\square$ from the van der Pauw measurements and areal electron density N_s from Hall measurements on the fabricated device, are used to obtain electron mobility μ. At temperature $T = 4.2$ K, it is found that $N_s = 3 - 3.4\times$ 10^{15} m^{-2} (depending on device), and $R_\square = 2.75$ $\Omega/\square$, yielding $\mu \approx 670 - 756$ $\mathrm{m}^2\mathrm{V}^{-1}\mathrm{s}^{-1}$ (confirming the cleanliness of the material) and momentum relaxing mean-free path $\ell_{\mathrm{MR}} = 65 - 69$ μm, calculated as $\ell_{\mathrm{MR}} = v_F\tau_{\mathrm{MR}}$. Here v_F denotes the Fermi velocity, with $v_F = 2.21 - 2.72\times 10^5$ m/s over the range $4.2\,\mathrm{K} < T < 36\,\mathrm{K}$, and τ_{MR} denotes the momentum relaxation time obtained from $\mu = e\tau_{\mathrm{MR}}/m$ with e the electron charge and $m = 0.067$ m_e the effective electron mass, with m_e the free electron mass. The Fermi energy, $E_F = 10.1 - 11.2$ meV for $4.2\,\mathrm{K} < T < 36\,\mathrm{K}$. Non-parabolicity of the band structure was taken into account in calculating the transport properties[2,3]. N_s (Supplementary Fig. 1a) and $R_\square$ (Supplementary Fig. 1b) increase with increasing T, while $\mu \sim 1/T$ (Supplementary Fig. 1c), as expected since μ is limited by scattering with acoustic phonons. Supplementary Figure 1d depicts $1/\mu$ vs T, indicating that $1/\mu(T) = 1/\mu_o + \alpha T$, where μ_o denotes μ limited by impurity scattering, α denotes a proportionality constant, and αT describes the linear dependence on T due to (predominantly) acoustic phonon consistent with theory in the equipartition regime (4-40 K)[4]. The rate of MR scattering in the equipartition regime is approximated as[4]:

$$\frac{1}{\tau_{\mathrm{MR}}} = \frac{1}{\tau_{ph}} + \frac{1}{\tau_{\mathrm{MR,o}}} \approx A_{ph}T + \frac{1}{\tau_{\mathrm{MR,o}}} \tag{1}$$

where $1/\tau_{ph}$ represents the phonon scattering rate, A_{ph} the phonon scattering coefficient, and $1/\tau_{\mathrm{MR,o}}$ the residual scattering rate due to impurities[5]. By plotting $1/\tau_{\mathrm{MR}}$ (with τ_{MR} calculated

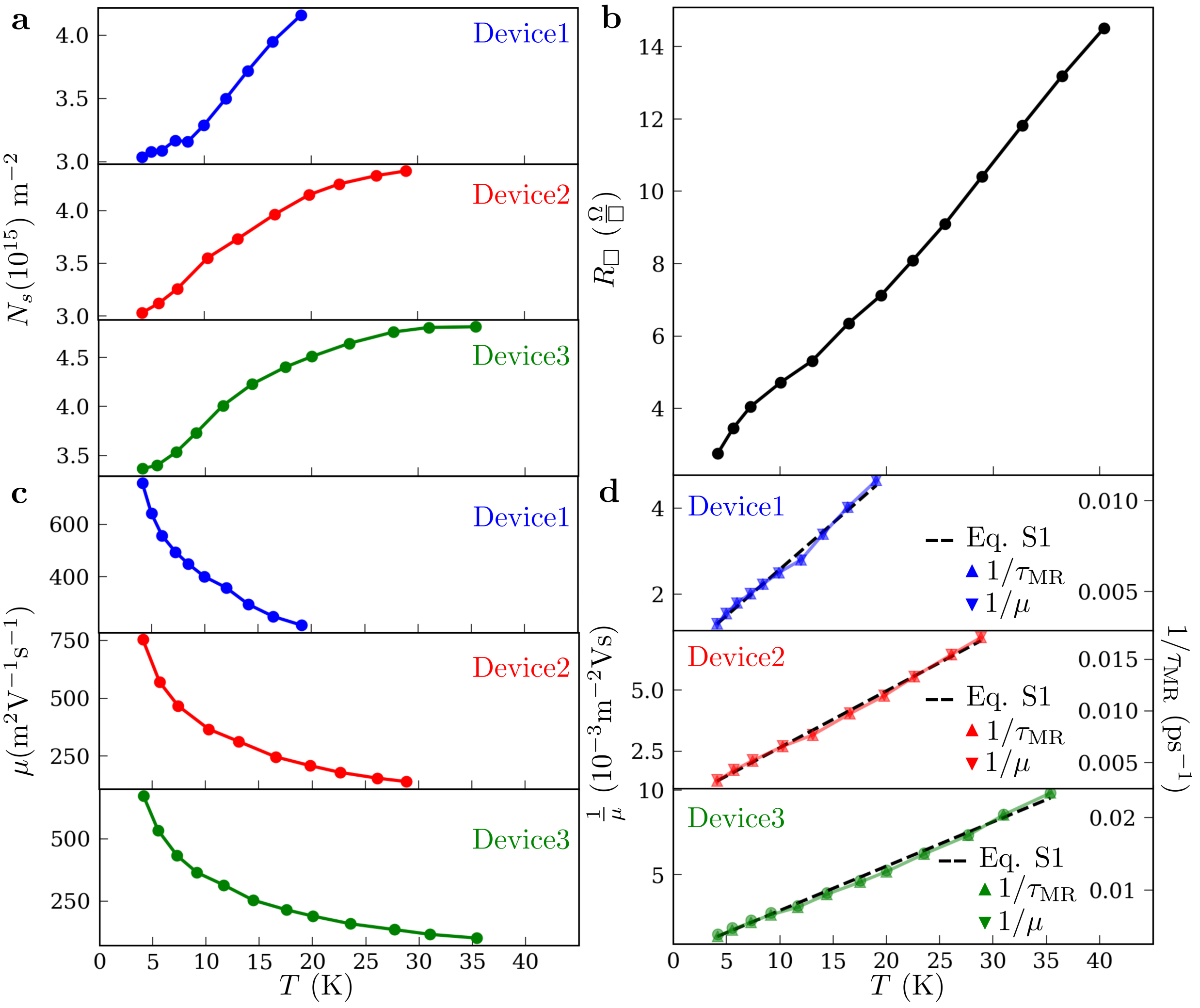

Supplementary Figure 1 | Transport characteristics. **a,** Carrier density N_s vs T for Device 1 (blue), Device2 (red) and Device3 (green). **b,** 2D resistivity $R_\square$ vs T from van der Pauw measurements on the unpatterned material. **c,** Mobility μ vs T. **d,** $1/\mu$ vs T (left axis) and $1/\tau_{\rm MR}$ vs T (right axis). From the linear fit (black dashed line) and Supplementary Eq. (1), we extract $A_{ph} \approx 5.1 - 6.2 \times 10^8$ s^{-1}K^{-1} and $\tau_{\rm MR,o} \approx 9.1 - 13.8 \times 10^{-10}$ s depending on the device.

from experimental values of $N_s(T)$ and $\mu(T)$ (Supplementary Fig. 1d), we indeed find that Supplementary Eq. (1) describes the dependence on T of $1/\tau_{\rm MR}$ well and that acoustic phonon scattering dominates the MR scattering in the range of T of the experiments, as expected for a high-μ 2DES.

Supplementary Note 2. Properties of TMF

Due to expected reciprocity relations[6,7], TMF results should be symmetric on exchanging the injector i and collector c and changing the polarity of magnetic field B applied normal to the 2DES

plane. Supplementary Figure 2a shows an example of this relation for Device1. While discrepancies can arise in mesoscopic devices[8], in the present devices these are small, allowing us to show the TMF spectra for only one polarity of B. The cyclotron diameter d_c approaches the conducting aperture width $w \approx 0.6\,\mu$m at $B = 0.3$ T, and hence well-defined semiclassical cyclotron orbits reflecting off the barrier require $B < 0.3$ T. As Supplementary Fig. 2b illustrates, the relevant data in Device1 hence occurs for $B \leq 0.2$ T (for other devices, the range of B can be different depending on L_c). Supplementary Figure 2b also illustrates the magnetoresistance background often superposed on TMF (dashed red line). The magnetoresistance background occurs independently of the TMF and can have several origins. The background can be carefully identified using smoothing filters (Supplementary Ref.[9] uses a Gaussian smoothing filter) and then be removed. In this work,

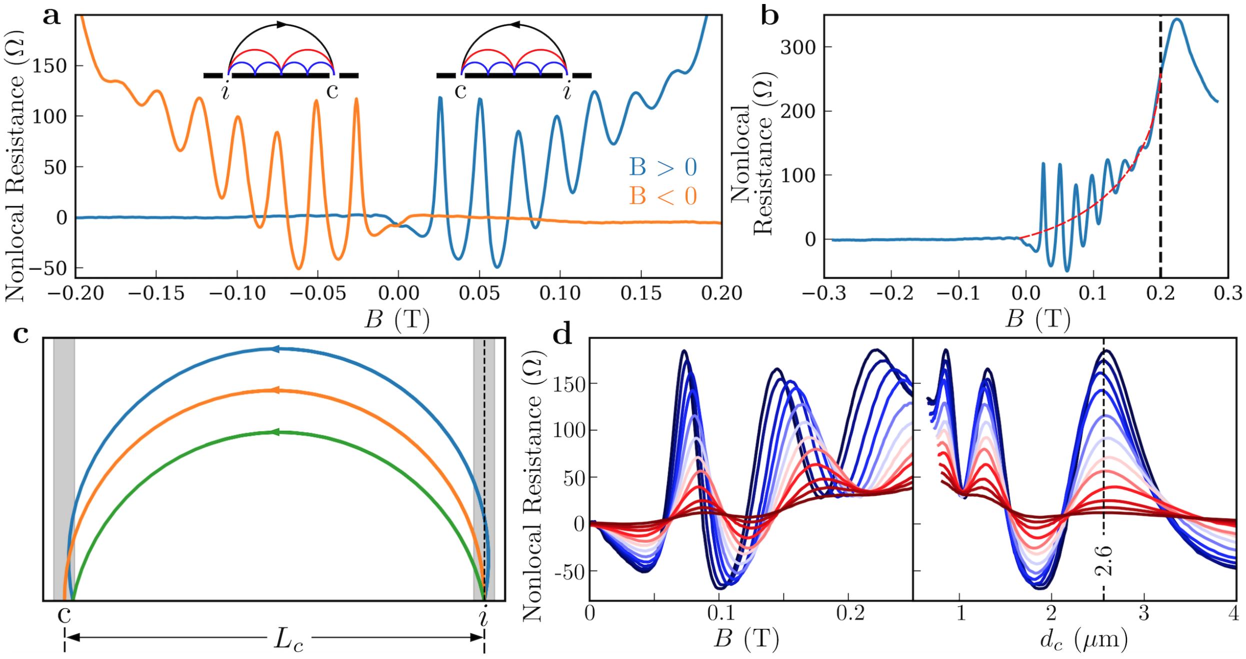

Supplementary Figure 2 | Properties of TMF. **a,** TMF at $T = 4.2$ K for D1 ($L_c = 7$ μm) when current and voltage contacts are exchanged and polarity of B inverted, illustrating reciprocity in resistance. The insets depict the semiclassical orbits ($\pi/2$ injection) corresponding to the TMF maxima. **b,** $B > 0$ spectra in (a) illustrating that the relevant data lies at $B < 0.2$ T. The dashed red line depicts the positive background magnetoresistance superposed on TMF. **c,** TMF geometry and cyclotron orbits, illustrated with the injection angle differing from $\pi/2$ to the barrier (orange orbit's injection angle at $\pi/2$ as reference). The grey vertical bars depict the injector and collector PC apertures. **d,** left panel: TMF spectra for Device3 ($L_c = 2.6$ μm) illustrating a shift vs B as T is increased. Right panel: the shift vanishes and the TMF spectra coincide when plotted vs d_c.

.

however, for one-to-one correspondence with the simulations, we do not remove the background and present the untreated experimental signal.

Supplementary Figure 2c illustrates that a cyclotron orbit starting at an angle different from $\pi/2$ to the barrier, will land in the vicinity of c at a distance less than $L = d_c$ from i (illustrated case corresponds to first maximum in the TMF spectrum). However, if the injection angles are not far from $\pi/2$, the orbits undergo magnetic focusing onto c, a property due to d_c being an extremal length scale of the cyclotron orbit for a GaAs 2DES. More generally, in a semiclassical approach, under B the path in reciprocal space coincides with cross-sections of the Fermi surface corresponding to equal-energy contours[10,11]. In real space the cyclotron orbit corresponds to a path of the same shape, rotated by $\pi/2$ and scaled as $1/B$. TMF spectra emphasize those orbits corresponding to extremal Fermi surface cross-sections. If the Fermi surface is circular with diameter $2k_F$ (as for a GaAs 2DES), then $d_c = 2\hbar k_F/eB$ (where k_F represents the Fermi wave vector). Carrier reflection from a potential barrier obtained by gentle wet etching is predominantly specular and hence TMF maxima appear when $L_c = nd_{cn} = 2\hbar k_F/eB_n$ where n is an integer and B_n is the magnetic field corresponding to the nth maximum (inset in Supplementary Fig. 2a). However, as discussed in the main text, due to spread in the injection angles, maxima will appear at B slightly lower than B_n. In Supplementary Figs. 2a-b, the TMF amplitude decreases with increasing $n > 3$ and for $n > 6$, the maxima lose definition. Two reasons contribute to this effect: 1) at higher B, $d_c \lesssim w$ and the semiclassical cyclotron orbits then do not have a clear point or angle of origin in the injector, and 2) as discussed in the main text, the spread in injection angles leads to a gradual defocusing after several skipping events off the barrier, even under perfect specular reflection off the barrier.

As illustrated in Fig. 3a main text, a shift in B in the TMF spectra and hence at the location of TMF maxima is observed as T is increased. The shift is due to increasing N_s with T (Supplementary Fig. 1a) leading to an increase in $k_F = \sqrt{2\pi N_s}$ with T. Since the position of c in the experiments remains fixed ($L_c = nd_{cn}$ remains fixed), $B_n = 2\hbar k_F/(e\, nd_{cn})$ increases proportionately with increasing k_F. Using $L_c = 2.6$ μm in Device3, Supplementary Fig. 2d illustrates that when the TMF specta are plotted vs B the shift is clearly visible, while the shift vanishes when the TMF spectra are plotted vs d_c.

As seen in Fig. 3a main text and Supplementary Fig. 6, the dependence of experimentally observed TMF peaks amplitude on n is non-monotonic, as has also been observed in other works[9,12–14]. While such non-monotonic behavior might result from a combination of various factors such as precise details of the boundary or the width of point contacts, we surmise that residual impurities incurred during the GaAs/AlGaAs growth might be playing an important part, even for the ul-

traclean material. Depending on n and d_c, certain electron orbits can elastically scatter at the randomly positioned residual impurities, which results in fewer electrons focusing at the collector as compared to for orbits which do not encounter the impurities. In the experiments this leads to a variation in individual TMF peak amplitudes with no systematic dependence on n. Another contributing factor to the variation in peak amplitudes can be the magnetoresistance background that the TMF peaks ride on which can make some TMF peaks appear larger than others.

Supplementary Note 3. Effects of ℓ_{MC} and d_c on the current streamlines and voltage profiles

Here we depict simulation results of the spatial current streamlines and voltage profiles on the theoretical test device T1 (5 μm × 5 μm square device), with current injection from the right edge and extraction from the left edge (Supplementary Fig. 3). We also depict additional simulation results of the current streamlines and voltage profiles on Device1 (Supplementary Fig. 4). The test devices T1 and T2 (10 μm × 7.5 μm rectangular device) help develop an intuition

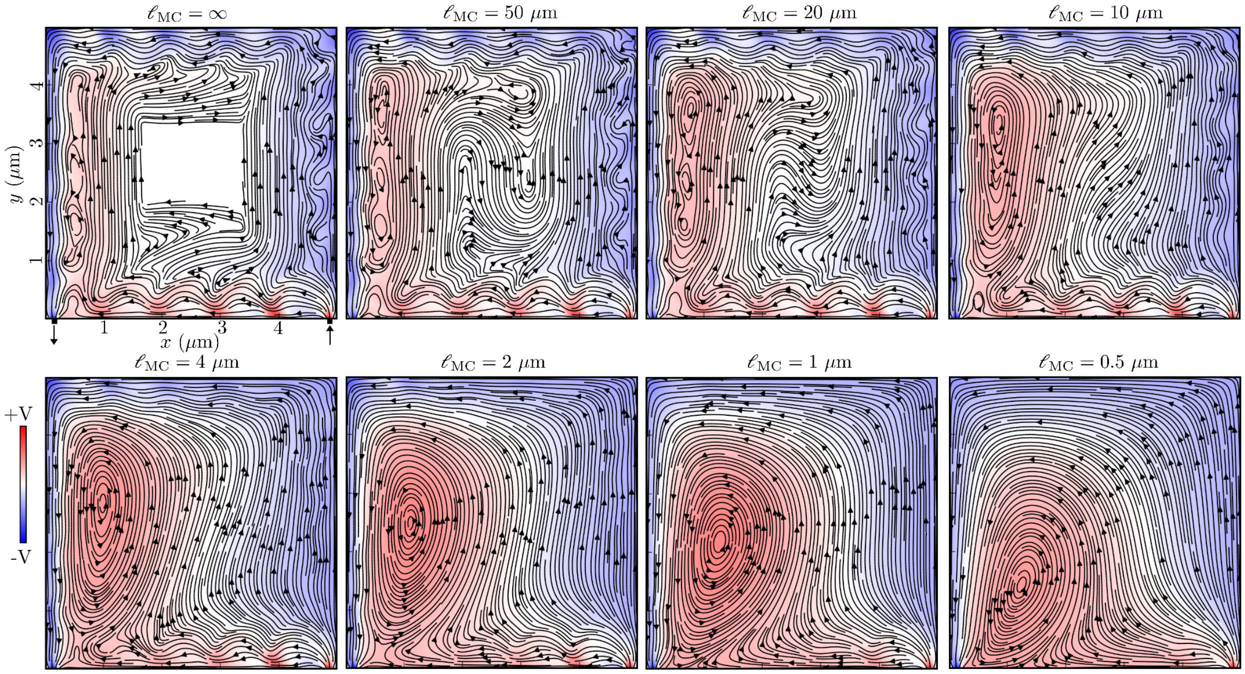

Supplementary Figure 3 | Effect of ℓ_{MC} on TMF. Current streamlines and voltage contour plots for T1 at several values of ℓ_{MC} with fixed $d_c = 1\ \mu$m. Starting with $\ell_{\mathrm{MC}} \to \infty$, we observe a ballistic flow profile with skipping cyclotron orbits propagating along the edges of the device. As ℓ_{MC} approaches the device scale, a vortex forms as expected when the system approaches the hydrodynamic regime (short ℓ_{MC}). When $\ell_{\mathrm{MC}} \lesssim d_c = 1\,\mu$m, the cyclotron orbits are suppressed by the device scale vortex. With ℓ_{MC} the shortest length scale, the system is then in the hydrodynamic regime.

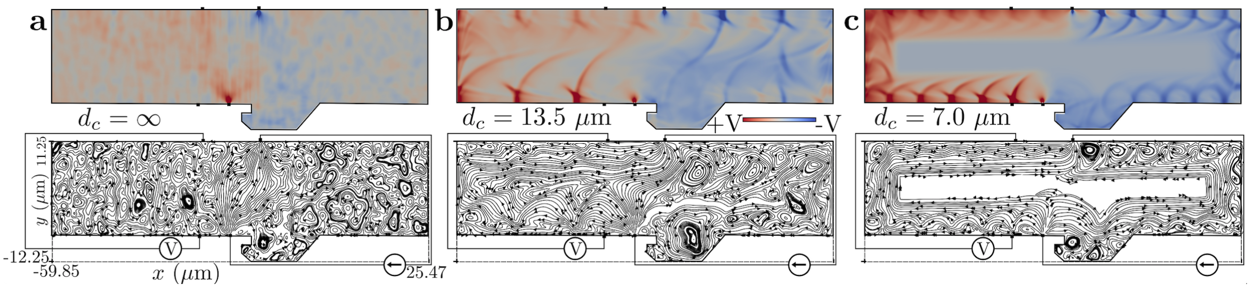

Supplementary Figure 4 | Effect of d_c (or B) on TMF a, Current streamline and voltage contour plots for Device1 at $B = 0$, where the device exhibits numerous current vortices forming at various device scales due to (specular) scattering at the device boundaries[15–17]. **b,** Current streamline and voltage contour plots for small $B = 0.013$ T, corresponding to $d_c = 13.5\ \mu$m. The current lines are more streamlined. Yet, since $d_c = 13.5\ \mu$m approaches the device scale $W \sim 21\ \mu$m, scattering at the device boundaries still influences the current streamlines and voltage contours, as discussed in Supplementary Note 4. **c,** Current streamline and voltage contour plots for $B = 0.025$ T, corresponding to $d_c = 7.0\ \mu$m. The local net current flows follow the device boundaries, minimizing scattering at boundaries.

for results in more complex experimental geometries, such as Device1. In Fig. 2b main text we depicted universal curves for a normalized $R_{n=1}$ vs parameter d_c/ℓ_{MC} for geometry T1 (for $L_c = d_c = 0.5,\ 1.0\,\mu$m; variable ℓ_{MC}), for geometry T2 (for $L_c = d_c = 1.5,\ 2.5\,\mu$m; variable ℓ_{MC}), and for Device1 ($L_c = d_c = 7\ \mu$m; variable ℓ_{MC}). We showed that the normalized curves for all the devices overlap, such that $R_{n=1}$ follows an exponential decay vs d_c/ℓ_{MC} independent of geometry.

In the simulations we set $\ell_{\mathrm{MR}} \to \infty$. In Supplementary Fig. 3 we visualize the effect of the variable ℓ_{MC} on the current streamlines and voltage profiles in T1 at a fixed $d_c = 1\,\mu$m (corresponding to the blue curve in Fig. 2b main text for $L_c = d_c = 1.0\,\mu$m). Salient observations are a ballistic flow profile with skipping cyclotron orbits along the edges of the device persisting for $\ell_{\mathrm{MC}} \gtrsim 4\,\mu$m, and a vortex forming and consuming the cyclotron orbits as ℓ_{MC} approaches first the device scale and then d_c (in the hydrodynamic regime). In Supplementary Fig. 4, we visualize the effect of variable d_c (hence variable B) on the current streamlines and voltage profiles in Device1 in the ballistic limit ($\ell_{\mathrm{MC}} \to \infty$). A higher B (lower d_c) encourages larger-scale features in the current streamlines, suppressing the wide range of sizes observed for current vortices at $B = 0$, ultimately tending to the local net current flows following the device boundaries.

Supplementary Note 4. Effect of $d_c \sim W$

A precondition for an accurate measurement of ℓ_{MC} from the TMF signal is $d_c < W$, where W represents the device scale. When $d_c \sim W$, (specular) boundary scattering affects the TMF signal (as observed in Supplementary Fig. 4b). The decay of $R_{n=1}$ with ℓ_{MC} then deviates from the universal curve, as mentioned in the main text. We exemplify this deviation in Supplementary Fig. 5 using $d_c = 2$ μm in the T1 device with $W = 5$ μm ($\ell_{\mathrm{MR}} \to \infty$ in the simulations). We note that the deviation is particularly noticeable near the ballistic limit ($\ell_{\mathrm{MC}} \to \infty$), where the dominant scattering occurs with device boundaries. In contrast, with strong MC scattering (short $\ell_{\mathrm{MC}} < W$), the deviation is subdued owing to ℓ_{MC} and not W being the shortest length scale in the system. We point out that despite this expected deviation from universality, one can still obtain values for α by justifiably not considering the deviant data points in the curve fitting.

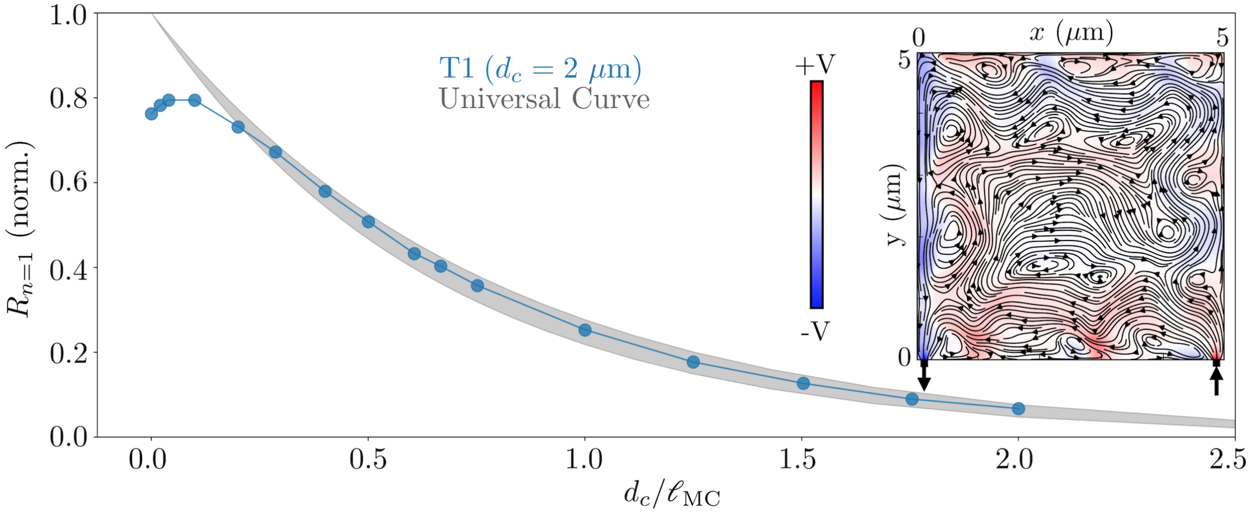

Supplementary Figure 5 | Effect of $d_c \sim$ W. $R_{n=1}$ vs d_c/ℓ_{MC} for $d_c = 2$ μm in geometry T1. The deviation from the universal curve (shaded grey region) at long ℓ_{MC} (close to the ballistic limit) can be attributed to boundary scattering effects originating from the condition $d_c \ll W$ being violated. The inset depicts the current streamlines and voltage contours at $d_c = 2$ μm for $\ell_{\mathrm{MC}} \to \infty$ (ballistic limit). Comparing to the first panel of Supplementary Fig. 3 (with $d_c = 1$ μm and $\ell_{\mathrm{MC}} \to \infty$) where the current flow is restricted to boundaries with little effect from parallel or perpendicular boundaries, a clear difference is visible with the boundaries affecting the current streamlines.

Supplementary Note 5. Experimental TMF spectra for remaining L_c

The hitherto undepicted experimental TMF spectra for distances L_c in Device2 and Device3, used for estimating $\ell_{\mathrm{MC}}(T_c)$ (Fig. 3c main text), are depicted in Supplementary Fig. 6. In Device2, L_c ranges from 3 μm to 15 μm with W= 42 μm. In Device3, L_c ranges from 1.3 μm to 20.5 μm with W= 24 μm. We omitted the longer L_c >12.8 μm in Device3 because of boundary effects as

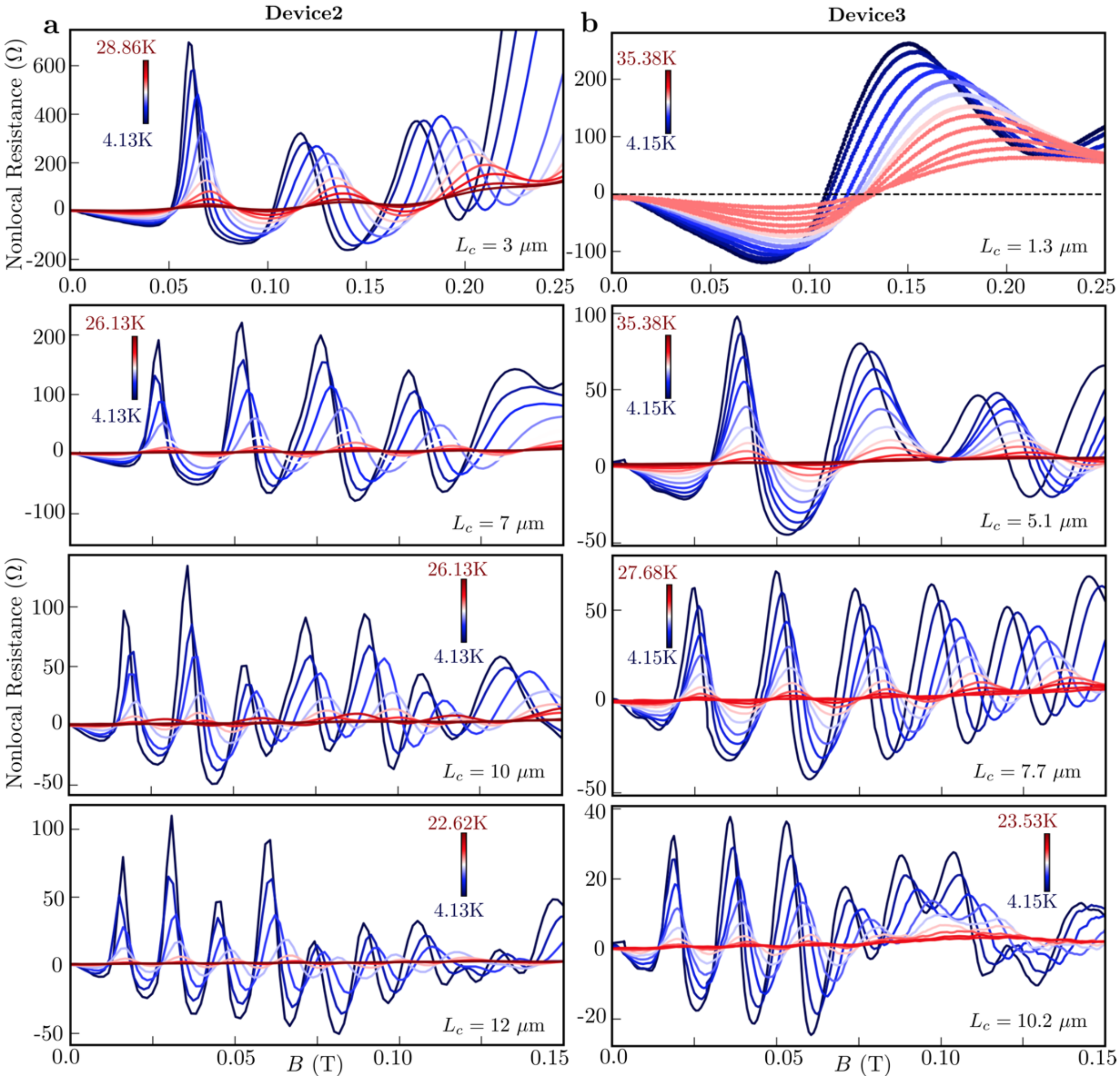

Supplementary Figure 6 | Experimental TMF spectra a, Experimentally measured TMF spectra for L_c = 3, 7, 10, 12 μm in Device2. **b,** Experimentally measured TMF spectra for L_c = 1.3, 5.1, 7.7, 10.2 μm in Device3. The range of T for each L_c is indicated. We observe an upward shift in B for the location of maxima with increasing T, due to increasing N_s (Supplementary Fig. 2d).

discussed in the previous section. Supplementary Note 2 (Supplementary Fig. 2d) discusses the observed upward shift in B for the location of maxima as T increases, due to increasing N_s.

Supplementary Note 6. A closed form expression for T_c

By eliminating the B dependent quantities $R_{n=1}(d_c, 0)$ and $\Delta_{n=1}(d_c)$ in Eq. (4) main text, we derive a closed form expression for T_c. Rewriting Eq. (4) with $R_{n=1}$ measured at $T = T + \delta T$, we obtain:

$$R_{n=1}(d_c, T + \delta T) = R_{n=1}(d_c, 0) - \Delta_{n=1}(d_c)\left(1 - \exp\left(-\left(\frac{T + \delta T}{T_c}\right)^2\right)\right) \tag{2}$$

Subtracting Eq. (4) main text from the above, we eliminate one of the non-universal parameters, $R_{n=1}(d_c, 0)$:

$$\begin{aligned}\delta R_{n=1}(d_c, T) &= R_{n=1}(d_c, T + \delta T) - R_{n=1}(d_c, T)\\ &= \Delta_{n=1}(d_c)\left(\exp\left(-\left(\frac{T + \delta T}{T_c}\right)^2\right) - \exp\left(-\left(\frac{T}{T_c}\right)^2\right)\right)\end{aligned} \tag{3}$$

Using the first order Taylor expansion, we write $\delta R_{n=1}(d_c, T)$ as:

$$\delta R_{n=1}(d_c, T) = -\Delta_{n=1}(d_c)\frac{2T\delta T}{T_c^2}\left(\exp\left(-\left(\frac{T}{T_c}\right)^2\right)\right) \tag{4}$$

Dividing $\delta R_{n=1}(d_c, T_1)$ and $\delta R_{n=1}(d_c, T_2)$ measured at two distinct temperatures $T = T_1$ and $T = T_2$, we eliminate the other non-universal parameter $\Delta_{n=1}(d_c)$:

$$\frac{\delta R_{n=1}(d_c, T_1)}{\delta R_{n=1}(d_c, T_2)} = \frac{T_1\delta T_1}{T_2\delta T_2}\left(\exp\left(\frac{T_2^2 - T_1^2}{T_c^2}\right)\right) \tag{5}$$

Rearranging and, taking the natural log on both sides,

$$\ln\left(\frac{\delta R_{n=1}(d_c, T_1)}{T_1\delta T_1}\frac{T_2\delta T_2}{\delta R_{n=1}(d_c, T_2)}\right) = \left(\frac{T_2^2 - T_1^2}{T_c^2}\right) \tag{6}$$

In the limit $\delta T \to 0$, we obtain the closed form expression for T_c:

$$T_c^2 = -\frac{T_2^2 - T_1^2}{\ln\left(\frac{1}{T}\mid\frac{\partial R_{n=1}}{\partial T}\mid\right)\Big|_{T_2} - \ln\left(\frac{1}{T}\mid\frac{\partial R_{n=1}}{\partial T}\mid\right)\Big|_{T_1}} \tag{7}$$

where $\partial R_{n=1}/\partial T$ is the temperature slope of $R_{n=1}$. Supplementary Equation (7) shows that in principle T_c can be obtained without fitting procedure. We note that for the Supplementary Eq. (7) to be valid, the experimental data must obey Eq. (4) main text, a necessary check that must be performed.

Supplementary Note 7. Fitting procedures and sources of uncertainty in the estimation of $\ell_{\mathbf{mc}}$

In Eq. (4) main text, $R_{n=1}(d_c, T = 0)$, $\Delta_{n=1}(d_c)$ and T_c are the three fitting parameters to the experimental $R_{n=1}(d_c, T)$ vs T data.

Uncertainties in the calculation of ℓ_{MC} using the method described in this work result from the following sources :

1. Uncertainty in the determination of the universal decay constant α, which could result from the following:
   (a) Curve fitting error: Error in the determination of α by fitting the simulated decay curve ($R_{n=1}(d_c, \ell_{\text{MC}})$ vs ℓ_{MC}) for a fixed geometry at fixed B while varying ℓ_{MC}, to Eq. (1) main text. This error can be reduced by using a large number of closely-spaced simulated data points in the decay curve, and ensuring that each simulation has run for a long enough time to reach steady state solution, both resulting in a better fit. The maximum fitting error is found to be 0.039 (obtained from simulation data in different device geometries under several representative B). We note that this uncertainty is smaller than the statistical uncertainty described below.
   (b) Statistical uncertainty: Spread in the estimation of α obtained by fitting Eq. (1) main text to $R_{n=1}(d_c, \ell_{\text{MC}})$ vs ℓ_{MC} curves obtained from simulations, each with different B and/or device geometries. As mentioned in the main text, the value and associated statistical uncertainty in the simulations are found as $\alpha = 1.34 \pm 0.1$.
   (c) Position of voltage counterprobe: We note that placing the voltage counterprobe ($V-$) too near the injector PC (where cyclotron orbits may impinge on it) can introduce a systematic error in the estimation of α. The universal value $\alpha = 1.34 \pm 0.1$ is found when the counterprobe is placed at a faraway location where the reference voltage remains constant as a function of T and B. In the simulated test geometries T1 and T2, we achieve this by keeping the counterprobe at zero potential, which is defined to be the potential at which the carrier density is the same as the unperturbed equilibrium

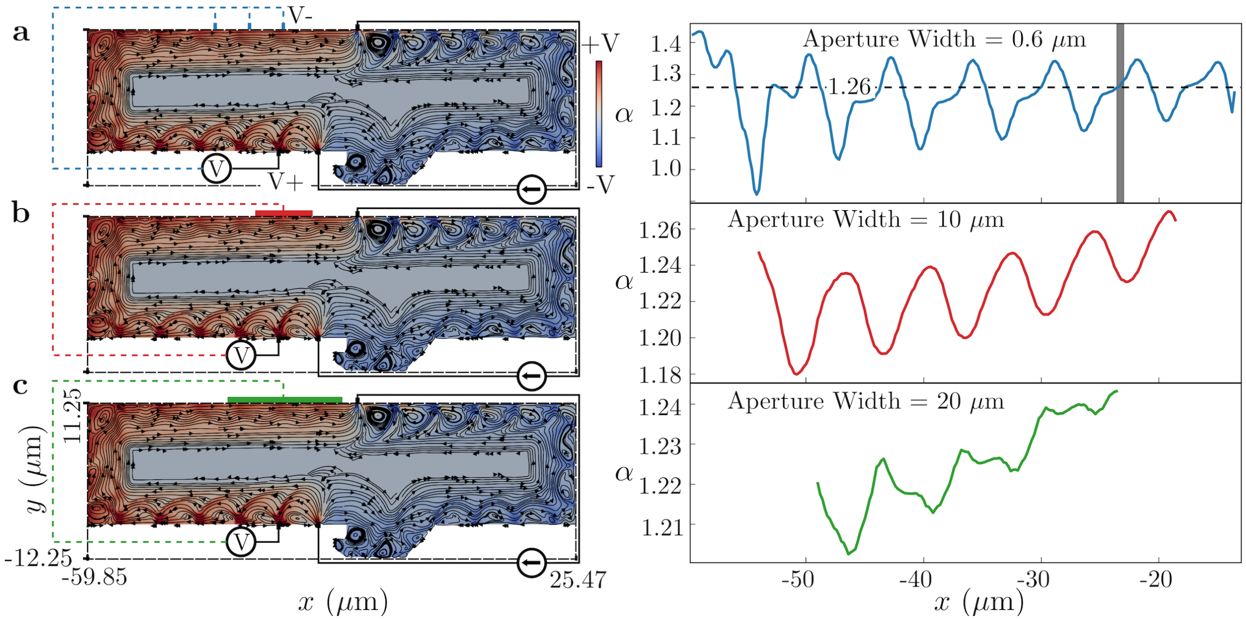

Supplementary Figure 7 | Variation of α with voltage counterprobe position. a,b,c. Current streamlines and voltage contour plots for Device1, when the position of the voltage counterprobe ($V-$), of aperture width **(a)** $0.6\,\mu$m, **(b)** $10\,\mu$m and **(c)** $20\,\mu$m, is varied along x on the top edge of the device (shown schematically with dotted blue lines in **(a)**). The right panels depict α vs the position x of the counterprobe for the corresponding counterprobe width in the left panel. The variation in α decreases on increasing the distance that skipping orbits originating from the injector have to travel along the boundary (counterclockwise in this case) to reach the counterprobe, and on increasing the width of the counterprobe aperture. The actual position of the counterprobe in the experiments is indicated by the grey bar in the right panel of **(a)**.

carrier density (see for example, white region in center of first panel in Supplementary Fig. 3). In Device1, for one-to-one correspondence with the experiments, we place the counterprobe in the simulations at the same location as in the experiments, which yields $\alpha = 1.26$ (inset of Fig. 2a main text). However, as illustrated in Supplementary Fig. 7, in Device1 we find that α varies as the position of the counterprobe is moved horizontally ($\| x$ in Supplementary Fig. 7) along the top edge of the device. The variation decreases as we move the counterprobe to the right owing to the carriers from the injector PC having to traverse a larger distance before impinging on the counterprobe (Supplementary Fig. 7a). We conclude that placing the counterprobe faraway from the injector PC (at a distance $\gg d_c$) leads to a lower error in the experimental estimate of α and hence of ℓ_{MC}. Another approach for minimizing the variation in α consists of increasing the conducting width of the counterprobe apertures, which averages out po-

tential variations within the counterprobe width (Supplementary Fig. 7b-c). We make use of both approaches in measurements on Device2 and Device3, where counterprobes of large width $\sim 20\,\mu$m are separated by up to mm (hence many d_c and many $\ell_{\rm MR}$) from the injector PCs.

2. Experimental curve fitting uncertainty: The maximal % uncertainty, across three devices, in the fitting parameters $R_{n=1}(d_c, T=0)$ and $\Delta_{n=1}(d_c)$ obtained by fitting Eq. (4) main text, to the experimental $R_{n=1}(d_c, T)$ vs T data, are 8.5% and 8.7% respectively. The uncertainty in fitting parameter T_c is less than 5.9%.

SUPPLEMENTARY REFERENCES

[1] Gardner, G. C., Fallahi, S., Watson, J. D. & Manfra, M. J. Modified MBE hardware and techniques and role of gallium purity for attainment of two dimensional electron gas mobility $> 35 \times 10^6$ cm^2/Vs in AlGaAs/GaAs quantum wells grown by MBE. *J. Crys. Growth*, **441**, 71-77 (2016).

[2] Zawadzki, W. & Szymanska, W. Elastic electron scattering in InSb-type semiconductors. *phys. stat. sol. (b)* **45**, 415 (1971).

[3] Zawadzki, W. Semirelativity in semiconductors: a review. *J. Phys. Condens. Matter* **29**, 373004 (2017).

[4] Harris, J. J. et al. Acoustic phonon scattering in ultra high mobility, low carrier density GaAs/(Al,Ga)As heterojunctions. *Surf. Sci.* **229**, 113-115 (1990).

[5] Lucas, A. & Fong, K. C. Hydrodynamics of electrons in graphene. *J. Phys. Condens. Matter* **30**, 053001 (2018).

[6] Büttiker, M. Four-terminal phase-coherent conductance. *Phys. Rev. Lett.* **57**, 1761 (1986).

[7] Büttiker, M. Symmetry of electrical conduction *IBM J. Res. Develop.* **32**, 317-334 (1988).

[8] Sánchez, D. & Kang K. Validity and breakdown of Onsager symmetry in mesoscopic conductors interacting with environment. *Phys. Rev. Lett.* **100**, 036806 (2008).

[9] Lee, M. et al. Ballistic miniband conduction in a graphene superlattice. *Science* **353**, 1526-1529 (2016).

[10] Tsoi, V. S. Focusing of electrons in a metal by a transverse magnetic field. *JETP Lett.* **19**, 70-71 (1974).

[11] Heremans, J. J., Santos, M. B. & Shayegan, M. Transverse magnetic focusing and dispersion of GaAs 2D holes at (311)A heterojunctions. *Surf. Sci.* **305**, 348-352 (1994).

[12] van Houten, H. et al. Coherent electron focusing with quantum point contacts in a two-dimensional electron gas. *Phys. Rev. B* **39**, 8556 (1989).

[13] Heremans, J. J., Santos, M. B. & Shayegan M. Observation of magnetic focusing in two-dimensional hole systems. *Appl. Phys. Lett.* **61**, 1652 (1992).

[14] Heremans, J. J., von Molnár, S., Awschalom, D. D. & Gossard, A. C. Ballistic electron focusing by elliptic reflecting barriers. *Appl. Phys. Lett.* **74**, 1281 (1999).

[15] Chandra, M., Kataria G., Sahdev, D. & Sundararaman, R. Hydrodynamic and ballistic AC transport in two-dimensional Fermi liquids. *Phys. Rev. B* **99**, 165409 (2019).

[16] Chandra, M., Kataria, G., & Sahdev, D. Quantum critical ballistic transport in two-dimensional Fermi liquids. Preprint at https://arxiv.org/abs/1910.13737 (2019).

[17] Gupta, A. et al. Hydrodynamic and ballistic transport over large length scales in GaAs/AlGaAs. *Phys. Rev. Lett.* **126**, 076803 (2021).
